# Kullback–Leibler Divergence Based Distributed Cubature Kalman Filter and Its Application in Cooperative Space Object Tracking

**DOI:** 10.3390/e20020116

**Published:** 2018-02-10

**Authors:** Chen Hu, Haoshen Lin, Zhenhua Li, Bing He, Gang Liu

**Affiliations:** 1 Xi’an Institute of High-Tech, Xi’an 710025, Shaanxi, China; 2 Institute of Electronics, Chinese Academy of Sciences, Beijing 100190, China

**Keywords:** cooperative space object tracking, distributed sensor network, distributed estimation, cubature Kalman filter, Kullback–Leibler divergence, consensus

## Abstract

In this paper, a distributed Bayesian filter design was studied for nonlinear dynamics and measurement mapping based on Kullback–Leibler divergence. In a distributed structure, the nonlinear filter becomes a challenging problem, since each sensor cannot access the global measurement likelihood function over the whole network, and some sensors have weak observability of the state. To solve the problem in a sensor network, the distributed Bayesian filter problem was converted into an optimization problem by maximizing a posterior method. The global cost function over the whole network was decomposed into the sum of the local cost function, where the local cost function can be solved by each sensor. With the help of the Kullback–Leibler divergence, the global estimate was approximated in each sensor by communicating with its neighbors. Based on the proposed distributed Bayesian filter structure, a distributed cubature Kalman filter (DCKF) was proposed. Finally, a cooperative space object tracking problem was studied for illustration. The simulation results demonstrated that the proposed algorithm can solve the issues of varying communication topology and weak observability of some sensors.

## 1. Introduction

Recently, space situational awareness (SSA) [1,2,3] has attracted more and more attention, because of its broad applications in space surveillance, tracking of objects in Earth orbit, monitoring the conditions of Earth’s magnetosphere, ionosphere and thermosphere, etc. Among the varieties of SSA systems, the space-based sensor network [4] (e.g., distributed satellite system) has many advantages compared with ground-based ones, since there is no atmosphere and weather problems for space-based sensor networks. Object tracking is a key problem in SSA, because many space missions highly depend on the results of object tracking, i.e., threat detection, cooperative search and collision avoidance. Kalman filter-based estimation [5,6] plays a key role among tracking methods, due to its ability of real-time estimation and non-stationary process tracking.

The main challenging problem in tracking objects in Earth orbit is the strong nonlinear dynamics of objects. Existing approximate nonlinear filters can be roughly classified into two categories: linearization and sampling approaches. The linearization approach is based on linearizing the nonlinear dynamics and measurement map and then employing the classical Kalman filter equations. For example, the extended Kalman filter (EKF) [7,8] puts the Jacobians of dynamics and measurement maps into the structure of the Kalman filter to estimate the state and corresponding covariance. It should be noted that the EKF is highly effective and has a broad range of applications [9,10]. The sampling approach is to use a collection of state samples to approximate the state estimate and its error covariance, i.e., uncertain Kalman filter (UKF) [11,12] and cubature Kalman filter (CKF) [13]. In [5], the authors studied the spacecraft tracking problem using sampled-data Kalman filters. Notice that UKF introduces a nonzero scaling parameter, which defines a non-zero center of sampling points. The CKF does not entail any free parameter and is more accurate than the UKF [14]. There exist many works studying the centralized estimation method, which need a central node to fuse information from the whole network [15,16]. For example, in [15], the authors proposed a fusion method based on the cubature information filter for the target tracking problem. However, when it comes to a distributed setting and the central node failure, information is exchanged only between neighbors.

Most of the existing distributed Kalman filters include consensus terms in the Kalman filter structure [17,18,19,20,21,22]. For example, Olfati-Saber [18] constructed a Kalman consensus filter (KCF), in which the estimates of each node are provided by consensus on measurement information. The work in [17] proposed an optimal KCF and then proved the convergence property. However, KCF in [17] is not scalable, since it needs all-to-all communications. Additionally, Zhou et al. [23,24] studied KCF for switching communication topologies. The work in [19] considered the KCF with consensus on the inverse covariance matrix and the information vector. It should be noted that the works in [19] only need communication once between two sample instances and do not need any global information. When it comes to the nonlinear case, the main difficulty is that the joint (all-sensor) likelihood function is not available for each sensor. Several distributed Bayesian filters were proposed to solve the problem [20,25,26]. In [25], a likelihood consensus-based distributed Bayesian filtering was studied, where the joint likelihood function was approximated by the consensus algorithm, and a distributed particle filter was proposed. Nevertheless, particle filters suffer from the burden of computational complexity, which is not suitable for real-time applications. In [20], the authors extended the results of [19] to a class of nonlinear systems and proposed a distributed extended Kalman filter. In [26], the authors proposed a distributed cubature information filter (DCIF), which was used for the cooperative space object tracking problem. However, in [26], information of the whole network is need, which may not be suitable for application. Therefore, we need to investigate a more scalable distributed Bayesian filter.

In this paper, a distributed sensor network architecture without a fusion center is considered, and a global estimation (global estimation means that the measurements of all sensors are processed by one sensor) task is performed by consensus algorithms through local processing and communicating with neighbors such that the final global estimate is obtained locally at each sensor. We first discuss the distributed Bayesian filter (DBF) by maximizing a posterior estimation method. Then K–L divergence-based consensus is used to approach the global posterior estimation. In order to improve the effectiveness and practicality of DBF, the cubature rules are adopted to formulate a distributed cubature Kalman filter. Notice that K–L divergence as an information metric has been used in several Kalman filters [19,27,28,29,30]. Similar to [19], K–L divergence in our paper is used to measure the difference of posterior distributions between sensors.

The contributions of this paper are summarized as follows:

1. A distributed Bayesian filter is developed, which can be treated as an extension of the traditional Bayesian filter [7] and an extension of distributed linear filters [18,19,31] to a nonlinear case. By maximizing a posterior estimation method, we show that the global posterior estimation can be achieved by consensus of each local posterior distribution, where the consensus of PDFs is obtained by an information-theoretic approach.
2. Based on the developed distributed Bayesian filter structure, a distributed cubature Kalman filter (DCKF) is proposed, which can improve the effectiveness and practicality for applications. Different from the design in [26], the only global information we required is the number of sensor, which is more suitable for applications.
3. The cooperative space object tracking problem is studied. Different from [26], we focus on the scenario in which the communication topology may change due to the blockage of the Earth. Moreover, we also consider the case that measurement mapping of each sensor may differ, which will lead to the problem of weak observability for some sensors. The issues of weak observability and blockage are handled by the proposed DCKF.

The remainder of paper is organized as follows. The problem formulation is given in Section 2. Then, the distributed Bayesian filter is discussed, while a fully-distributed cubature Kalman filter is proposed in Section 3. Following that, numerical simulations on space object tracking are shown in Section 4. The discussions are provided in Section 5. Finally, the conclusion of this paper is provided in Section 6.

Notations: The superscript “⊤” represents the transpose. E{x} denotes the mathematical expectation of the stochastic variable *x*. diag{·} represent the diagonalization scalar elements. tr(P) is the trace of the matrix *P*, and var(x) is the variance of *x*. p(·) denotes the probability density function (PDF), and N(0,U) is the Gaussian distribution with mean zero and variance matrix *U*.

## 2. Problem Formulation

In this section, we first give the formulating of distributed Bayesian filtering and describe the consensus of PDFs.

### 2.1. Distributed Bayesian Filter Formulation

Consider the following discrete-time stochastic non-linear dynamics,

(1) $$\begin{array}{c} x_{k+1}=f\left(x_{k}\right)+w_{k}, \end{array}$$

where $x_{k}\in R^{n}$ is the state that needs to be estimated and $w_{k}$ is zero mean Gaussian noise with $E\left\{w_{k}w_{k}^{⊤}\right\}=Q_{k}$. Dynamics (1) describes the state transition $p(x_{k}|x_{k-1})$. Assume that the state $x_{k}$ is observed by a network of *N* sensors, whose measurement model is given as:

(2) $$\begin{array}{c} z_{i,k}=h\left(x_{k}\right)+v_{i,k},\;i=1,2,\ldots ,N, \end{array}$$

where $z_{i,k}\in R^{m_{i}}$ is the measurement by sensor *i* at time *k*. $v_{i,k}∼N\left(0,R_{i,k}\right)$ is the measurement noise of sensor *i*, where $0\in R_{i}^{m}$ denotes the zero vector. Measurement Equation (2) describes the measurement likelihood function $p(z_{i,k}|x_{k}),\;i=1,2,\ldots ,N$. In this paper, we assume that $w_{k}$ and $v_{i,k}\forall i\in V$ are independent, and all $z_{i,k},\forall i\in V$ are conditionally independent of $x_{k}$.

The communication of the network is modeled by an undirected graph $G_{k}=\left(V,E_{k},A_{k}\right)$, which consists of the set of sensors V={1,2,…,N}, the set of edges $E_{k}\subseteq V\times V$ and the weighted adjacent matrix $A_{k}=\left[a_{ij,k}\right]$. In the weighted adjacent matrix $A_{k}$, all the elements are nonnegative, row stochastic, and the diagonal elements are all positive, i.e., $a_{ii,k}>0,a_{ij,k}\geq 0,\sum_{j\in V}a_{ij,k}=1$. If $a_{ij,k}>0,j\neq i$, there is an edge $\left(i,j\right)\in E_{k}$, which means nodes *i* and *j* can directly communicate, and node *j* is called the neighbor of node *i*. The degree matrix is defined as $D^{\left(G\right)}=diag\left\{d_{1},d_{2},\cdots ,d_{N}\right\}\in R^{N\times N}$, where the diagonal element $d_{i}$ is the number of nodes connected to node *i*. All the neighbors of node *i* including itself can be represented by the set $\left\{j\in V|\left(i,j\right)\in E_{k}\right\}⋃\left\{i\right\}≜N_{i,k}$, whose size is denoted as $|N_{i,k}|$. In this paper, we assume that the undirected graph $G_{k}$ is connected for all *k*.

The adjacent matrix $A_{k}$ represents the weights of nodes. Note that for a certain graph, there exists an infinite number of associated adjacency matrices. To ensure the double stochastic nature of adjacent matrix $A_{k}$, a possible choice of the weights [32] is:

(3) $$\begin{array}{cc} a_{ij,k} & =\frac{1}{\max\{|N_{i,k}|,|N_{j,k}|\}},\;j\in N_{i,k},i\neq j, \end{array}$$

(4) $$\begin{array}{cc} a_{ii,k} & =1-\sum_{j\in N_{i,k},j\neq i}a_{ij,k}. \end{array}$$

Denote the global measurement as $z_{k}={\left[z_{1,k}^{⊤},z_{2,k}^{⊤},\ldots ,z_{N,k}^{⊤}\right]}^{⊤}$. The relationship between $z_{k}$ and $x_{k}$ can be given as global likelihood function $p(z_{k}|x_{k})$, and the relationship between $x_{k}$ and measurement $z_{i,k}$ can be described as local likelihood function $p(z_{i,k}|x_{k})$. Under the assumption that the agents are independent of each other, the global likelihood function can be expressed as a product of local likelihood functions,

(5) $$\begin{array}{c} p\left(z_{k}|x_{k}\right)=\prod_{i=1}^{N}p\left(z_{i,k}|x_{k}\right). \end{array}$$

In this paper, we assume that the state $x_{k}$ is conditional independent with all past measurements $z_{1:k-1}$, i.e., $p\left(x_{k}|z_{1:k}\right)=p\left(x_{k}|z_{k}\right)$. Sensor *i* knows the dynamics (1) and local likelihood function $p(z_{i,k}|x_{k})$ and does not know the global likelihood function $p(z_{k}|x_{k})$. Sensor *i* can only communicate with its neighbors.

The aim of the Bayesian filter is to compute posterior distribution $p(x_{k}|z_{k})$. The recursive solution to compute $p(x_{k}|z_{k})$ consists of prediction and update steps. The predictive distribution of state $x_{k}$ can be given by the Chapman–Kolmogorov equation,

(6) $$\begin{array}{c} p\left(x_{k}|z_{k-1}\right)=\int p\left(x_{k}|x_{k-1}\right)p\left(x_{k-1}|z_{k-1}\right)dx_{k-1}. \end{array}$$

The posterior can be given as:

(7) $$\begin{array}{c} p\left(x_{k}|z_{k}\right)=\frac{1}{\tilde{c}}p\left(x_{k}|z_{k-1}\right)p\left(z_{k}|x_{k}\right), \end{array}$$

where $\tilde{c}=\int p\left(x_{k}|z_{k-1}\right)p\left(z_{k}|x_{k}\right)dx_{k}$ denotes the normalization. However, for the Bayesian filter (6) and (7), the computational complexity of state estimation is usually intractable. A computationally-feasible approximation is provided by the cubature Kalman filter [13,14], which uses cubature rules to compute numerical integration for multi-dimensional integrals. It has been shown that the CKF has better performance compared with EKF and UKF [13].

It can be seen from (6) and (7) that if one can access the global likelihood $p(z_{k}|x_{k})$, the global estimate ${\hat{x}}_{k}$ can be obtained. However, in our paper, each sensor only knows local likelihood function $p(z_{i,k}|x_{k})$, and therefore, we have to propose a distributed approach to estimate $x_{k}$. In Section 3, we proposed a distributed Bayesian filter based on the consensus of PDFs, which is obtained by the Kullback–Leibler divergence described in Section 2.2.

### 2.2. Consensus of Probability Densities

In this section, we will describe the consensus of probability density functions, which will be used to solve the distributed Bayesian filter problem in Section 3.

The traditional average consensus problem is defined under Euclidean space [32]. However, the measure under Euclidean space is not suitable for the probability distribution. For example, two normal distributions N(0, 10,000) and N(10, 10,000) are almost indistinguishable, the Euclidean distance between the parameter is 10. In contrast, the distributions N(0,0.01) and N(0.1,0.01) barely overlap, but this is not reflected in the Euclidean distance, which is only 0.1. A more natural measure between two densities is Kullback–Leibler divergence rather than Euclidean distance.

K–L divergence between two PDFs p(·) and q(·) is defined as:

(8) $$\begin{array}{c} D_{KL}\left(p∥q\right)=\int p(x)\log\frac{p(x)}{q(x)}dx. \end{array}$$

Following [19,33], the centroid depending on K–L divergence (CKL) is considered, which describes the centroid form initial PDFs,

(9) $$\begin{array}{c} \bar{p}=\arg\inf\sum_{i=1}^{N}a_{i}D_{KL}\left(p∥p_{i}\right). \end{array}$$

where $a_{i}\geq 0,\forall i$ are weights and should satisfy $\sum_{i\in N}a_{i}=1$. The centroid in (9) turns out to be [19],

(10) $$\begin{array}{c} \bar{p}\left(x\right)=\frac{\prod_{i=1}^{N}{\left[p_{i}\left(x\right)\right]}^{a_{i}}}{\int \prod_{i=1}^{N}{\left[p_{i}\left(x\right)\right]}^{a_{i}}dx}. \end{array}$$

It is worth noting that the CKL can be seen as an example of Bregman information as the mean of Bregman divergence [34,35]. An important feature of (10) is that it is suitable for distributed computation. Namely, the CKL can be achieved by some consensus algorithms, which requires that the data are only transmitted between agents and their neighbors at each step. Thus, CKL can be computed by the consensus algorithm as follows,

(11) $$\begin{array}{c} p_{i}^{\left(t\right)}\left(x\right)=\frac{\prod_{j\in N_{i}}{\left[p_{j}^{\left(t-1\right)}\left(x\right)\right]}^{a_{ij,k}}}{\int \prod_{j\in N_{i}}{\left[p_{j}^{\left(t-1\right)}\left(x\right)\right]}^{a_{ij,k}}dx} \end{array}$$

where t=1,2,… refer to the *t*-th step and $a_{ij,k}$ is the weights between agent *i* and *j*.

When the distribution is given, the consensus of PDFs will be achieved by manipulating the corresponding parameters. The following lemma shows how to compute consensus on the exponential family iteratively, which can be found in [36].

**Lemma** **1.** *Consider the network $G_{k}=\left(V,E_{k},A_{k}\right)$. Let a PDF $p_{i}^{\left(t\right)}\left(x\right)=f\left(x;\lambda_{i}^{\left(t\right)}\right),\forall i\in N$ be exponential distribution families, where λ is a natural parameter. Then, iteratively update (11) given by:*

(12) $$\begin{array}{c} \lambda_{i}^{\left(t+1\right)}=\sum_{j\in N_{i}}a_{ij,k}\lambda_{j}^{\left(t\right)} \end{array}$$

**Remark** **1.** *An exponential family can be expressed in the following form [37],*

(13) $$\begin{array}{c} p\left(\theta \right)=h\left(\theta \right)\exp\{\lambda^{⊤}u\left(\theta \right)-A_{g}\left(\lambda \right)\}, \end{array}$$

*where λ is a natural parameter, $A_{g}\left(\lambda \right)$ is a log-normalizer and h(θ) is a carrier measure [38]. The exponential families include many of the most common distributions, e.g., Gaussian, Poisson, Bernoulli, Wishart, and many other. Namely, those distributions can be written in the form of exponential families (13).*

**Remark** **2.** It should be noted that K–L divergence defined in (8) is not symmetric. In [33], the authors discussed sided Bregman centroids, i.e., right-sided centroid ${\bar{p}}^{R}=\arg\inf\sum_{i=1}^{N}a_{i}D_{KL}\left(p_{i}∥p\right)$ and left-sided centroid ${\bar{p}}^{L}=\arg\inf\sum_{i=1}^{N}a_{i}D_{KL}\left(p∥p_{i}\right)$. As shown in [33] (Theorem 3.1 in [33]), ${\bar{p}}^{R}$ can be expressed as a convex combination of PFDs, i.e., ${\bar{p}}^{R}=\sum_{i=1}^{N}a_{i}p_{i}$, which always is the center of mass. Notice that ${\bar{p}}^{R}$ is hard to obtain, if $p_{i}$ and $p_{j}$ are correlated. However, for the distributed estimation problem, $p_{i}$ from different nodes need to be fused at each time k, and consequently, they are correlated (see [17]). In this paper, we only consider the left-sided centroid (9), which is easy to compute as shown in (12).

## 3. Distributed Cubature Kalman Filter

In this section, we first discuss the distributed Bayesian filter based on the maximum a posterior method and propose a distributed cubature Kalman filter based on K–L divergence.

### 3.1. Distributed Bayesian Filter

The global posterior distribution can be given as:

(14) $$\begin{array}{cc} p(x_{k}|z_{k}) & =\frac{1}{\tilde{c}}p\left(x_{k}|z_{k-1}\right)p\left(y_{k}|x_{k}\right) \end{array}$$

(15) $$\begin{array}{c} \;=\frac{1}{\tilde{c}}p\left(x_{k}|z_{k-1}\right)\prod_{i=1}^{N}p\left(z_{i,k}|x_{k}\right). \end{array}$$

Notice that the predictive distribution is $p\left(x_{k}|z_{k-1}\right)=N\left({\tilde{x}}_{k},{\tilde{P}}_{k}\right)$, and the likelihood function is $p\left(z_{i,k}|x_{k}\right)=N\left(z_{i,k}-h_{i}\left(x_{k}\right)|R_{i,k}\right)$. Therefore, under the assumption of Gaussian noises, we can obtain the log posterior distribution as follows,

(16) $$\begin{array}{cc} \log p(x_{k}|z_{k})= & \log\frac{1}{\tilde{c}}+\log p\left(x_{k}|z_{k-1}\right)+\sum_{i=1}^{N}\log p\left(z_{i,k}|x_{k}\right)\\ = & \log\frac{1}{\tilde{c}}+\log\frac{1}{\sqrt{{\left(2\pi \right)}^{n}\left|{\tilde{P}}_{k}\right|}}+\sum_{i=1}^{N}\log\frac{1}{\sqrt{{\left(2\pi \right)}^{m_{i}}|R_{i,k}}|} \end{array}$$

(17) $$\begin{array}{c} \;-\frac{1}{2}{\left(x_{k}-{\tilde{x}}_{k}\right)}^{⊤}{\tilde{P}}_{k}^{-1}\left(x_{k}-{\tilde{x}}_{k}\right)-\frac{1}{2}\sum_{i=1}^{N}{\left(z_{i,k}-h_{i}\left(x_{k}\right)\right)}^{⊤}R_{i,k}^{-1}\left(z_{i,k}-h_{i}\left(x_{k}\right)\right). \end{array}$$

Rearranging the items of Equation (17), we obtain:

(18) $$\begin{array}{c} \log p\left(x_{k}|z_{k}\right)=\tilde{C}+\frac{1}{N}\sum_{i=1}^{N}\left\{-\frac{1}{2}{\left(x_{k}-{\tilde{x}}_{k}\right)}^{⊤}{\tilde{P}}_{k}^{-1}\left(x_{k}-{\tilde{x}}_{k}\right)-\frac{1}{2}N{\left(z_{i,k}-h_{i}\left(x_{k}\right)\right)}^{⊤}R_{i,k}^{-1}\left(z_{i,k}-h_{i}\left(x_{k}\right)\right)\right\} \end{array}$$

where $\tilde{C}$ is a constant term that does not effect the estimate of $x_{k}$. By the maximum a posteriori method, our problem becomes:

(19) $$\begin{array}{c} \max_{x_{k}}\;\;F_{k}\left(x_{k}\right)=\frac{1}{N}\sum_{i=1}^{N}-f_{i,k}\left(x_{k}\right) \end{array}$$

where $f_{i,k}=\frac{1}{2}{\left(x_{k}-{\tilde{x}}_{k}\right)}^{⊤}{\tilde{P}}_{k}^{-1}\left(x_{k}-{\tilde{x}}_{k}\right)+\frac{1}{2}N{\left(z_{i,k}-h_{i}\left(x_{k}\right)\right)}^{⊤}R_{i,k}^{-1}\left(z_{i,k}-h_{i}\left(x_{k}\right)\right)$. Notice that Problem (19) is equivalent to $\min_{x_{k}}\;\frac{1}{N}\sum_{i=1}^{N}f_{i,k}\left(x_{k}\right)$.

Although the global cost function $F_{k}\left(x_{k}\right)$ over the whole network can be decomposed, we cannot independently minimize the local cost function $f_{i,k}\left(x_{k}\right)$ at each node to reach a global optimum. A key point is that the global cost function $-F_{k}\left(x_{k}\right)$ of the full measurements over the whole network is definitely larger than or equal to the average local cost function $f_{i,k}\left(x_{k}\right)$ over all nodes. Namely,

(20) $$\begin{array}{cc} \min_{x_{k}}\;-F_{k}\left(x_{k}\right) & =-F_{k}\left(x_{k}^{*}\right)\\ & =\frac{1}{N}\sum_{i=1}^{N}f_{i,k}\left(x_{k}^{*}\right)\\ & \geq \frac{1}{N}\sum_{i=1}^{N}f_{i,k}\left(x_{i,k}^{*}\right)\\ & =\frac{1}{N}\sum_{i=1}^{N}\min_{x_{k}}f_{i,k}\left(x_{k}\right), \end{array}$$

where $x_{k}^{*}$ is the optimal distribution minimizing $-F(x_{k})$ and $x_{k}^{*}$ is the one minimizing $f_{i,k}\left(x_{k}\right)$. The equality in the second line holds if and only if $x_{k}^{*}$ is also the optimal solution for all the local cost functions $f_{i,k}\left(x_{k}\right)$, which is not always the case. Therefore, we cannot find the optimal solution by individually minimizing the local cost function at each sensor. However, from (20), we can see that the global optimal solution over the whole network can be approximated by the average local optimal solution of each sensor. Therefore, we can construct a distributed approach to solve the problem based on average consensus.

Taking the derivative of $f_{i,k}$ with respect to $x_{k}$, we have:

(21) $$\begin{array}{cc} {▿}_{x_{k}}f_{i,k} & \approx {\tilde{P}}_{k}^{-1}\left(x_{k}-{\tilde{x}}_{k}\right)+NH_{i,k}^{⊤}R_{i,k}\left[h_{i}\left({\tilde{x}}_{k}\right)-z_{i,k}+H_{i,k}x_{k}-H_{i,k}{\tilde{x}}_{k}\right] \end{array}$$

(22) $$\begin{array}{c} \;=({\tilde{P}}_{k}^{-1}+NH_{i,k}^{⊤}R_{i,k}^{-1}H_{i,k})\left(x_{k}-{\tilde{x}}_{k}\right)+NH_{i,k}^{⊤}R_{i,k}^{-1}\left[h_{i}\left({\tilde{x}}_{k}\right)-z_{i,k}\right]. \end{array}$$

Here, we use the fact $h_{i}\left(x_{k}\right)\approx h_{i}\left(\tilde{x}\right)+H_{i,k}\left(x_{k}-{\tilde{x}}_{k}\right)$ with $H_{i,k}=\frac{\partial h_{i}\left(x_{k}\right)}{\partial x_{k}}{|}_{x_{k}={\tilde{x}}_{k}}$. Denote ${\overset{ˇ}{x}}_{i,k}$ as the optimal solution with respect to problem $\min_{x_{k}}\;f_{i,k}$. The estimate ${\overset{ˇ}{x}}_{i,k}$ by sensor *i* can be obtained by letting ${▿}_{x_{k}}f_{i,k}$ be equal to zero, and we get:

(23) $$\begin{array}{c} {\overset{ˇ}{x}}_{i,k}={\tilde{x}}_{k}+N{\left({\tilde{P}}_{k}^{-1}+NH_{i,k}^{⊤}R_{i,k}^{-1}H_{i,k}\right)}^{-1}H_{i,k}^{⊤}R_{i,k}^{-1}\left(z_{i,k}-h_{i}\left({\tilde{x}}_{k}\right)\right). \end{array}$$

By the matrix inverse lemma, we have:

(24) $$\begin{array}{cc} N{\left({\tilde{P}}_{k}^{-1}+NH_{i,k}^{⊤}R_{i,k}H_{i,k}\right)}^{-1}H_{i,k}^{⊤}R_{i,k}^{-1}= & {\left(I+N{\tilde{P}}_{k}H_{i,k}^{⊤}R_{i,k}H_{i,k}\right)}^{-1}N{\tilde{P}}_{k}H_{i,k}^{⊤}R_{i,k}^{-1} \end{array}$$

(25) $$\;\begin{array}{cc} = & N{\tilde{P}}_{k}H_{i,k}^{⊤}{\left(NH_{i,k}\tilde{P}H_{i,k}^{⊤}+R_{i,k}\right)}^{-1}. \end{array}$$

Substituting (25) into (23), we obtain:

(26) $$\begin{array}{c} {\overset{ˇ}{x}}_{i,k}={\tilde{x}}_{k}+N{\tilde{P}}_{k}H_{i,k}^{⊤}{\left(NH_{i,k}\tilde{P}H_{i,k}^{⊤}+R_{i,k}\right)}^{-1}\left(z_{i,k}-h_{i}\left({\tilde{x}}_{k}\right)\right). \end{array}$$

The estimate error covariance can be computed as follows,

(27) $$\begin{array}{c} {\overset{ˇ}{P}}_{i,k}={\tilde{P}}_{k}+P_{i,xz,k}P_{i,zz,k}^{-1}P_{i,xz,k}^{⊤}, \end{array}$$

where:

(28) $$\begin{array}{cc} P_{i,zz,k} & =NH_{i,k}\tilde{P}H_{i,k}^{⊤}+R_{i,k}, \end{array}$$

(29) $$\begin{array}{cc} P_{i,xz,k} & =N{\tilde{P}}_{k}H_{i,k}^{⊤}. \end{array}$$

**Remark** **3.** It should be noted that $P_{i,zz,k}$ and $P_{i,xz,k}$ are a little different with the standard extended Kalman filter, even though they can be obtained by each sensor individually. With N=1 in (26) and (27), it will reduce to the standard Kalman filter.

Up to now, we obtain the optimal solution of $\min_{x_{k}}\;f_{i,k}$ as ${\overset{ˇ}{x}}_{i,k}$ and ${\overset{ˇ}{P}}_{i,k}$, which follows the Gaussian distribution $N({\overset{ˇ}{x}}_{i,k},{\overset{ˇ}{P}}_{i,k})$. As discussed in (20), the global optimal solution can be approximated by averaging local estimate $N({\overset{ˇ}{x}}_{i,k},{\overset{ˇ}{P}}_{i,k})$. However, the traditional average consensus algorithm in Euclidean space may not be suitable to compute the average of PDFs. Therefore, we use the consensus of PDFs described in Section 2.2 to compute the global solution to Problem (19).

The natural parameter of Gaussian distribution $p_{i}\left(x|{\overset{ˇ}{x}}_{i},{\overset{ˇ}{P}}_{i}\right)$ is $\lambda_{i}=\left[\begin{array}{c} {\overset{ˇ}{P}}_{i}^{-1}{\overset{ˇ}{x}}_{i}\\ -\frac{1}{2}{\overset{ˇ}{P}}_{i}^{-1} \end{array}\right]$ [38]. Then, the global posterior distribution can be approximated by the consensus of probability densities (12) as follows,

(30) $$\begin{array}{c} {\left({\overset{ˇ}{P}}_{i}^{s+1}\right)}^{-1}{\overset{ˇ}{x}}_{i}^{s+1}=\sum_{j\in N_{i}}a_{ij,k}{\left({\overset{ˇ}{P}}_{j}^{s}\right)}^{-1}{\overset{ˇ}{x}}_{j}^{s}, \end{array}$$

(31) $$\begin{array}{c} {\left({\overset{ˇ}{P}}_{i}^{s+1}\right)}^{-1}=\sum_{j\in N_{i}}a_{ij,k}{\left({\overset{ˇ}{P}}_{i}^{s}\right)}^{-1}, \end{array}$$

where s=1,…,S is the step of the consensus. Then, the estimates of each node can be achieved by $P_{i,k}={\left({\overset{ˇ}{P}}_{i,k}^{S}\right)}^{-1}$ and ${\hat{x}}_{i,k}={\left(P_{i,k}\right)}^{-1}{\left({\overset{ˇ}{P}}_{i}^{S}\right)}^{-1}{\overset{ˇ}{x}}_{i}^{S}$.

**Remark** **4.** In [19], each sensor performed a standard Kalman filter, then the fusion estimation was obtained based on consensus of PDFs. It should be noted that, if N=1, (26)–(27) will reduce to the measurement update of the standard Kalman filter. We derive an optimal solution of each sensor for Problem (19), which may achieve better performance compared to [19]. However, we should highlight that [19] provided meaningful information the theoretical expression for the distributed filter.

Equations (26)–(31) provide a general framework of the distributed Bayesian filter for posterior estimation. Based on the measurement update Equations (26)–(31), we can construct distributed nonlinear filtering by combining the existing method for state propagation. For example, in [29], the ensemble Kalman filter (EnKF) was used for state propagation, which uses the Monte Carlo technique for integral operation in the Bayesian filtering. In this paper, we use cubature rules for state propagation and measurement update, which we will discuss in the following.

### 3.2. Distributed Cubature Kalman Filter

Suppose that the state $x_{k-1}$ is approximated by sensor *i* at time k−1 as follows,

(32) $$\begin{array}{c} p\left(x_{k-1}|z_{i,k-1}\right)=N\left({\hat{x}}_{i,k-1},{\hat{P}}_{i,k-1}\right), \end{array}$$

where N(x,P) denotes the Gaussian distribution with mean *x* and covariance *P*. The predictive distribution $p\left(x_{k}|z_{i,k-1}\right)=N\left({\tilde{x}}_{i,k},{\tilde{P}}_{i,k}\right)$ can be obtained by the prediction step of CKF. To be specific, a set of cubature points [13] can be provided by:

(33) $$\begin{array}{cc} X_{i,t,k-1}= & S_{i,k-1}\xi_{t}+{\hat{x}}_{i,k-1}, \end{array}$$

(34) $$\begin{array}{cc} X_{i,t,k}^{*}= & f(X_{i,t,k-1}), \end{array}$$

where the basic cubature point is given by $\xi_{t}=\sqrt{\frac{m}{2}}\times {\left[1\right]}_{t}$, t=1,…,m, $m=2n_{x}$ and ${\left[1\right]}_{t}$ denotes the *t*-th element of set [1]. For example, let $\left[1\right]\in R^{2}$, then it represents the set $\left\{\left[\begin{array}{c} 1\\ 0 \end{array}\right],\left[\begin{array}{c} 0\\ 1 \end{array}\right],\left[\begin{array}{c} -1\\ 0 \end{array}\right],\left[\begin{array}{c} 0\\ -1 \end{array}\right]\right\}$. Then, the predicted state and covariance are given by:

(35) $$\begin{array}{cc} {\tilde{x}}_{i,k}= & \frac{1}{m}\sum_{t=1}^{m}X_{i,t,k}^{*}, \end{array}$$

(36) $$\begin{array}{cc} {\tilde{P}}_{i,k}= & \frac{1}{m}\sum_{t=1}^{m}X_{i,t,k}^{*}X_{i,t,k}^{*⊤}-{\tilde{x}}_{i,k}{\tilde{x}}_{i,k}^{⊤}+Q_{k-1}. \end{array}$$

Denote ${\tilde{P}}_{i,k}={\tilde{S}}_{i,k}{\tilde{S}}_{i,k}^{⊤}$; under the assumption that these errors can be well approximated by the Gaussian, the prediction measurement can be obtained as follows,

(37) $$\begin{array}{c} {\tilde{z}}_{i,k}=\frac{1}{m}\sum_{t=1}^{m}{\tilde{Z}}_{i,t,k}. \end{array}$$

where the set of cubature points ${\tilde{Z}}_{i,t,k},t=1,\ldots ,m$ is given by:

(38) $$\begin{array}{cc} {\tilde{X}}_{i,t,k}= & {\tilde{S}}_{i,k}\xi_{t}+{\tilde{x}}_{i,k}, \end{array}$$

(39) $$\begin{array}{cc} {\tilde{Z}}_{i,t,k}= & h_{i}\left({\tilde{X}}_{i,t,k}\right). \end{array}$$

In the Bayesian framework, these prediction means and covariances will be incorporated in the procedure as prior information of the state to propel the measurement update. Based on Equations (26) and (27), the local posterior can be given as follows,

(40) $$\begin{array}{cc} {\overset{ˇ}{x}}_{i,k}= & {\tilde{x}}_{i,k}+P_{i,k,xz}P_{i,k,zz}^{-1}\left(z_{i,k}-{\tilde{z}}_{i,k}\right) \end{array}$$

(41) $$\begin{array}{cc} {\overset{ˇ}{P}}_{i,k}= & {\tilde{P}}_{k}+P_{i,k,xz}P_{i,k,zz}^{-1}P_{i,k,xz}^{⊤}. \end{array}$$

Different from the standard cubature Kalman filter, the innovation covariance matrix $P_{i,zz,k}$ and cross-covariance matrix $P_{i,xz,k}$ of node *i* can be given according to (28) and (29) as follows,

(42) $$\begin{array}{cc} P_{i,zz,k}= & N\left[\frac{1}{m}\sum_{t=1}^{m}{\tilde{Z}}_{i,t,k}{\tilde{Z}}_{i,t,k}^{⊤}-{\tilde{z}}_{i,k}{\tilde{z}}_{i,k}^{⊤}\right]+R_{i,k}, \end{array}$$

(43) $$\begin{array}{cc} P_{i,xz,k}= & N\left[\frac{1}{m}\sum_{t=1}^{m}{\tilde{X}}_{i,t,k}{\tilde{Z}}_{i,t,k}^{⊤}-{\tilde{x}}_{i,k}{\tilde{z}}_{i,k}^{⊤}\right]. \end{array}$$

By the consensus of Gaussian distributions (30), the global estimate can be approximated as:

(44) $$\begin{array}{c} {\left(P_{i}^{s+1}\right)}^{-1}{\hat{x}}_{i}^{s+1}=\sum_{j\in N_{i}}a_{ij,k}{\left(P_{j}^{s}\right)}^{-1}{\hat{x}}_{j}^{s},\;\;\;s=1,2,\ldots \end{array}$$

(45) $$\begin{array}{c} {\left(P_{i}^{s+1}\right)}^{-1}=\sum_{j\in N_{i}}a_{ij,k}{\left(P_{i}^{s}\right)}^{-1},\;\;\;s=1,2,\ldots . \end{array}$$

With the iterations (44) and (45), we can get the final estimation of state in each sensor. Meanwhile, the iterative estimation will approximate to the global solution of Problem (20) because of the convergence of the average consensus of PDFs as S→∞. In practice, the convergence will not be achieved fully, because the total number of iterations *S* is finite. Therefore, the distributed implementation will not perform as well as the centralized one. We summarize the distributed cubature Kalman filter in Algorithm 1.

In (36), $Q_{k}$ can be chosen as a sufficiently small constant matrix. Notice that the initialization of all the local estimates is exactly the same mean of the initial state. However, in practice, it is not easy to let every sensor know the prior knowledge. A more suitable setting is $x_{i,0}=0,\forall i\in V$ and $P_{i,0}=0,\forall i\in V$, which means that there does not exist prior knowledge.

In [26], the authors proposed DCIF for cooperative space object tracking. For comparison, we briefly summarize the main steps of DCIF in Table 1, where the prediction step is omitted due to it being the same as the prediction step of DCKF. In DCIF, ${\tilde{z}}_{i,k}$ and $P_{i,xz,k}$ can be obtained by (39) and (43), and $0<\epsilon <\frac{1}{\Delta_{max}}$, $\Delta_{max}=\max_{i}\left\{d_{i}\right\}$.

**Algorithm 1** DCKF at node *i* at time *k***Ensure:** At time *k*, a prior information $P_{i,k-1}=S_{i,k-1}S_{i,k-1}^{⊤}$ and ${\hat{x}}_{i,k-1}$;Prediction$$\begin{array}{cc} X_{i,t,k-1}= & S_{i,k-1}\xi_{t}+{\hat{x}}_{i,k-1},\;\;X_{i,t,k}^{*}=f\left(X_{i,t,k-1}\right),\;\;{\tilde{x}}_{i,k}=\frac{1}{m}\sum_{t=1}^{m}X_{i,t,k}^{*},\\ {\tilde{P}}_{i,k}= & \frac{1}{m}\sum_{t=1}^{m}X_{i,t,k}^{*}X_{i,t,k}^{*⊤}-{\tilde{x}}_{i,k}{\tilde{x}}_{i,k}^{⊤}+Q_{k-1}. \end{array}$$Local estimation–Measurement prediction
$$\begin{array}{c} {\tilde{z}}_{i,k}=\frac{1}{m}\sum_{t=1}^{m}{\tilde{Z}}_{i,t,k},\;\;{\tilde{X}}_{i,t,k}={\tilde{S}}_{i,k}\xi_{t}+{\tilde{x}}_{i,k},\;\;{\tilde{Z}}_{i,t,k}=h_{i}\left({\tilde{X}}_{i,t,k}\right). \end{array}$$–Local estimate and estimate error covariance
$$\begin{array}{cc} P_{i,zz,k}= & N\left[\frac{1}{m}\sum_{t=1}^{m}{\tilde{Z}}_{i,t,k}{\tilde{Z}}_{i,t,k}^{⊤}-{\tilde{z}}_{i,k}{\tilde{z}}_{i,k}^{⊤}\right]+R_{i,k},\\ P_{i,xz,k}= & N\left[\frac{1}{m}\sum_{t=1}^{m}{\tilde{X}}_{i,t,k}{\tilde{Z}}_{i,t,k}^{⊤}-{\tilde{x}}_{i,k}{\tilde{z}}_{i,k}^{⊤}\right],\\ {\overset{ˇ}{x}}_{i,k}= & {\tilde{x}}_{i,k}+P_{i,k,xz}P_{i,k,zz}^{-1}\left(z_{i,k}-{\tilde{z}}_{i,k}\right),\\ {\overset{ˇ}{P}}_{i,k}= & {\tilde{P}}_{k}+P_{i,k,xz}P_{i,k,zz}^{-1}P_{i,k,xz}^{⊤}. \end{array}$$Consensus–**for** s=1 to *S* **do**
$$\begin{array}{cc} {\left({\overset{ˇ}{P}}_{i}^{s+1}\right)}^{-1}{\overset{ˇ}{x}}_{i}^{s+1} & =\sum_{j\in N_{i}}a_{ij,k}{\left({\overset{ˇ}{P}}_{j}^{s}\right)}^{-1}{\overset{ˇ}{x}}_{j}^{s},\;\;\;s=1,2,\ldots \\ {\left({\overset{ˇ}{P}}_{i}^{s+1}\right)}^{-1} & =\sum_{j\in N_{i}}a_{ij,k}{\left({\overset{ˇ}{P}}_{i}^{s}\right)}^{-1},\;\;\;s=1,2,\ldots \end{array}$$–**end for**Compute the estimate ${\hat{x}}_{i,k}$ and covariance matrix $P_{i,k}$,
$$\begin{array}{c} P_{i,k}={\left({\overset{ˇ}{P}}_{i,k}^{S}\right)}^{-1},\;\;{\hat{x}}_{i,k}={\left(P_{i,k}\right)}^{-1}{\left({\overset{ˇ}{P}}_{i}^{S}\right)}^{-1}{\overset{ˇ}{x}}_{i}^{S}. \end{array}$$

**Remark** **5.** The algorithm in [26] can approach the centralized solution, which is achieved by performing a consensus on information pairs, i.e., ${\tilde{H}}_{i,k}^{⊤}R_{i,k}^{-1}\left({\overset{ˇ}{z}}_{i,k}+{\tilde{H}}_{i,k}{\tilde{x}}_{i,k}\right)$ and ${\tilde{H}}_{i,k}^{⊤}R_{i,k}^{-1}{\tilde{H}}_{i,k}$, where ${\tilde{H}}_{i,k}\approx {\overset{ˇ}{P}}_{i,k}^{-1}P_{i,xz,k}$ is the pseudo measurement matrix and ${\overset{ˇ}{z}}_{i,k}=z_{i,k}-{\tilde{z}}_{i,k}$. The main limitation of [26] is that it needs a sufficiently large number of consensus steps at each time step, so that the local information pairs can spread to the whole network. It should be noted that we do not limit the range of S. In [19], the convergence properties were proven for such a fusion principle, even S=1 in the linear dynamics case, which used the fact that the whole posterior PDFs are combined rather than the state or the information pairs. A distributed extended Kalman filter was discussed in [20], which needs the linearization of nonlinear dynamics and measurement mapping. We proposed a distributed cubature Kalman filter, which does not need linearization and can achieve better performance. On the other hand, we provide a structure for the distributed Bayesian filter with the help of K–L divergence, which could approach the centralized solution more efficiently compared with the one in Euclidean space.

**Remark** **6.** An important feature of proposed algorithm is that the only used global information is the number of sensors N, which is suitable in application. In [26], the author proposed a distributed cubature information filter (DCIF) for the cooperative tracking space object, where the number of sensor N and the maximum degree $\Delta_{\max}$ of the network are needed. However, in practice, $\Delta_{\max}$ may change and is not easy to obtain in time.

## 4. Numerical Simulations

**Figure 1:**
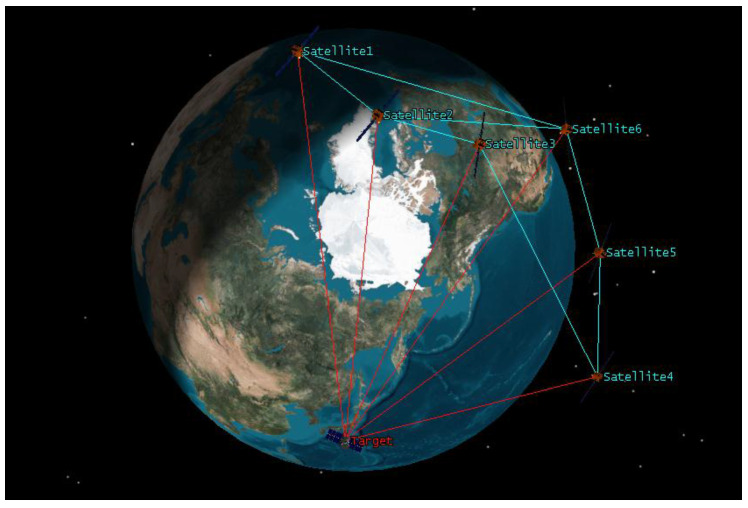
Scenario of cooperative space object tacking.

In this section, we illustrate the effectiveness of the proposed DCKF for the space object tracking problem, where the scenario is shown in Figure 1. A distributed satellite system is observing a non-cooperative object, where the bearing-only measurement information is considered. The number of observation satellites is N=6.

In what follows, we first give the dynamics of the space object and measurement mapping by a distributed satellite system, then we solve the cooperative space object tracking problem by the proposed DCKF.

### 4.1. Dynamics of Space Target

The dynamics of space object can be described as follows,

(46) $$\begin{array}{c} \ddot{r}=-\frac{\mu }{∥r{∥}^{3}}r+J_{2}+w, \end{array}$$

where $r={\left[rx\;ry\;rz\right]}^{⊤}$ represents the position of the object in the Earth-centered inertial (ECI) coordinate frame, μ is the gravitational constant, $J_{2}$ stands for perturbations and *w* is Gaussian noise with zero mean.

Denote $x={\left[rx,ry,rz,\dot{rx},\dot{ry},\dot{rz}\right]}^{⊤}$ as the state variables; we can rewrite (46) in state-space description as follows,

(47) $$\begin{array}{c} \dot{x}=\left[\begin{array}{c} \dot{rx}\\ \dot{ry}\\ \dot{rz}\\ -\frac{\mu }{∥r{∥}^{3}}rx+J_{2,1}+w_{1}\\ -\frac{\mu }{∥r{∥}^{3}}ry+J_{2,2}+w_{2}\\ -\frac{\mu }{∥r{∥}^{3}}rz+J_{2,3}+w_{3} \end{array}\right]=f\left(x\right), \end{array}$$

where $w={\left[w_{1},w_{2},w_{3}\right]}^{⊤}$ is process noise, and the perturbation has the following form,

(48) $$\begin{array}{c} J_{2}=\frac{3}{2}a_{J}{\left(\frac{E_{r}}{∥r{∥}^{3}}\right)}^{2}\frac{\mu }{∥r{∥}^{3}}\left[\begin{array}{c} rx(5\frac{rz^{2}}{∥r{∥}^{2}}-1)\\ ry(5\frac{rz^{2}}{∥r{∥}^{2}}-1)\\ rz(5\frac{rz^{2}}{∥r{∥}^{2}}-3) \end{array}\right], \end{array}$$

where $E_{r}$ is the Earth radius, $a_{J}\approx 0.00108263$.

Dynamics (47) is a continuous time model, thus (47) should be discretized in order to apply the EKF algorithm. Let $T=t_{k+1}-t_{k}$ be the sampling period, then the discrete model of (47) is described in the following,

(49) $$\begin{array}{c} x_{k+1}=x_{k}+\int_{t_{k}}^{t_{k+1}}f\left(x\left(t\right)\right)dt. \end{array}$$

When $T=t_{k+1}-t_{k}$ is sufficient small, the Taylor expansion of f(x(t)) is:

(50) $$\begin{array}{c} f\left(x\left(t\right)\right)\approx f\left(x_{k}\right)+A\left(x_{k}\right)f\left(x_{k}\right)\left(t-t_{k}\right), \end{array}$$

where $A(x_{k})$ has the following form:

(51) $$\begin{array}{c} A\left(x_{k}\right)=\frac{\partial f(x_{k})}{\partial x}{|}_{t=t_{k}}=\left[\begin{array}{cc} \frac{\partial \dot{r}}{\partial r} & \frac{\partial \dot{r}}{\partial \dot{r}}\\ \frac{\partial \ddot{r}}{\partial r} & \frac{\partial \ddot{r}}{\partial \dot{r}} \end{array}\right]=\left[\begin{array}{cc} 0_{3\times 3} & I_{3}\\ A_{21} & 0_{3\times 3} \end{array}\right]. \end{array}$$

Combine (49)–(51), the discrete model can be given as:

(52) $$\begin{array}{c} x_{k+1}=x_{k}+f\left(x_{k}\right)T+A\left(x_{k}\right)f\left(x_{k}\right)\frac{T^{2}}{2}, \end{array}$$

where $x_{k}\in R^{n_{x}}$ is the state that needs to be estimated.

The discretized dynamics (52) is used in the prediction step of the EKF. It can be seen that the higher order terms are ignored in (50), which may enlarge the estimate errors. The state of the object is completely represented by $x\left(t\right)\in R^{6}$, which includes its position and velocity. When we describe the satellite moving along an orbit, it is often represented in the form of six orbital parameters, i.e., the semi-major axis $a_{h}$, the eccentricity *e*, the inclination *u*, the argument of perigee γ, the longitude of the ascending node Γ and the mean anomaly $m_{h}$. The nonlinear transformations that converts position and velocity into orbital elements can be found in [39].

### 4.2. Measurement Model

The dynamics (47) is measured by a distributed satellites system. In this example, we consider a satellite equipped with an optical sensor that can obtain the azimuth α or elevation β of the object. Measurement mapping of azimuth α and elevation β can be expressed as follows,

(53) $$\begin{array}{cc} \alpha_{k} & =\arctan\left(\frac{ry_{k}-{\overset{ˇ}{ry}}_{k}}{rx_{k}-{\overset{ˇ}{rx}}_{k}}\right)+va_{k}, \end{array}$$

(54) $$\begin{array}{cc} \beta_{k} & =\arctan\left(\frac{rz_{k}-{\overset{ˇ}{rz}}_{k}}{\sqrt{{\left(rx_{k}-{\overset{ˇ}{rx}}_{k}\right)}^{2}+{\left(ry_{k}-{\overset{ˇ}{ry}}_{k}\right)}^{2}}}\right)+vb_{k}, \end{array}$$

where ${\left[{\overset{ˇ}{rx}}_{k},{\overset{ˇ}{ry}}_{k},{\overset{ˇ}{rz}}_{k}\right]}^{⊤}$ is the position of the satellite at time *k* and $va_{k}$ and $vb_{k}$ are measurement noise at time *k*.

In [26], the authors assume that the measurement equation of different satellites is the same. However, in practice, this does not always hold. In this example, we assume that a satellite can obtain either or both of the azimuth α and elevation β, which has a broader range of application. The measurement equation of the *i*-th satellite can be written as follows,

(55) $$\begin{array}{c} z_{i,k}=h_{i}\left(x_{k}\right)+v_{i,k},i=1,\ldots ,N, \end{array}$$

where *N* is the number of satellites. Due to blockage of the Earth, it is more suitable to model communication topology as time-varying.

**Figure 2:**
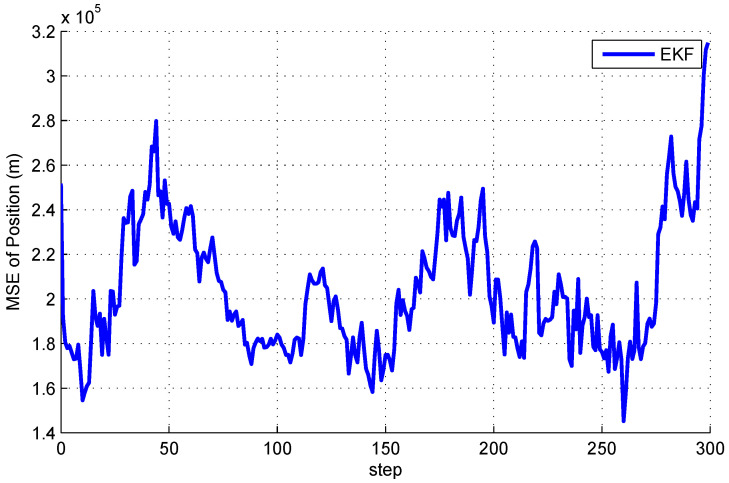
Mean square errors of position by a single satellite.

**Figure 3:**
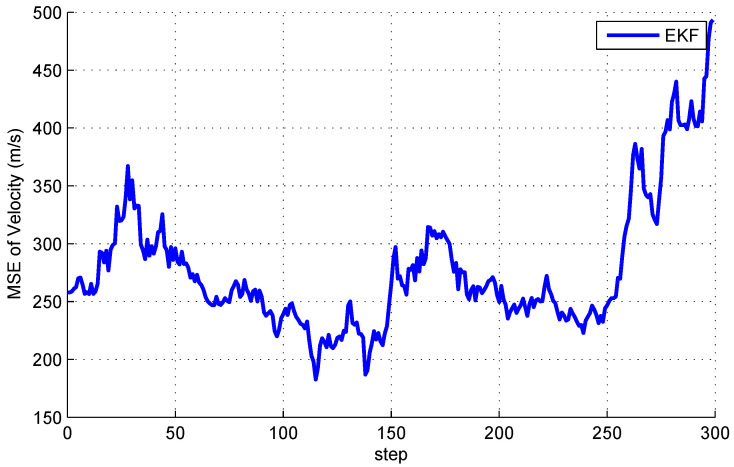
Mean square errors of velocity by a single satellite.

Our aim is to estimate state $x_{k}$ by a network of satellites. It should be noted that, if one satellite can only obtain azimuth α, then the estimates obtained only by this satellite will be very large or even divergent. Figure 2 and Figure 3 show the mean square error (MSE) of EKF for space object tacking in a single satellite, where only the azimuth α can be measured. It can be seen from Figure 2 and Figure 3 that both the MSE of position and velocity are very large. More importantly, since the Earth may block the communication between satellites, the communication topology may change. Therefore, it is not reasonable to know the information of the global communication topology for each satellite in real time.

### 4.3. Simulation Results

The trajectories of observation satellites and the true object are generated by the six orbital parameters as shown in Table 2. We consider the dynamics (47); the state of the object is $x={\left[rx,ry,rz,\dot{rx},\dot{ry},\dot{rz}\right]}^{⊤}$. The noise variance of the process is $Q_{k}=diag\left\{10^{-4},10^{-4},10^{-4},10^{-6},10^{-6},10^{-6}\right\}$. The initial state of each agent was chosen randomly from $N(x_{0},P_{0})$, where $x_{0}={\left[x_{p0}^{⊤}\;\;x_{v0}^{⊤}\right]}^{⊤}$ is the true initial state of the object, $P_{0}=diag\left[\left(10^{10}\;10^{10}\;10^{10}\;100^{2}\;100^{2}\;100^{2}\right)\right]$, and $x_{p0}$ = [$4.36\times 10^{6}$ m $3.13\times 10^{6}$ m $6.61\times 10^{6}$ m]${}^{⊤}$, $x_{v0}$ = [−5505.2 m/s −207.5 m/s 3954.8 m/s]${}^{⊤}$.

Numerical simulations are conducted through Monte Carlo experiment, in which 50 Monte Carlo trials are done for each tracking algorithm. The total of the mean square estimation errors (MSE) is considered, which is widely used to indicate the performance of estimates, which is defined as:

(56) $$\begin{array}{c} MSE_{k}=\frac{1}{N}\sum_{i=1}^{N}\left[\frac{1}{50}\sum_{j=1}^{50}{\left({\hat{x}}_{i,k}^{\left(j\right)}-x_{k}\right)}^{T}\left({\hat{x}}_{i,k}^{\left(j\right)}-x_{k}\right)\right]. \end{array}$$

The performance of mean square estimation for each satellite is defined as:

(57) $$\begin{array}{c} MSE_{i,k}=\frac{1}{50}\sum_{j=1}^{50}{\left({\hat{x}}_{i,k}^{\left(j\right)}-x_{k}\right)}^{T}\left({\hat{x}}_{i,k}^{\left(j\right)}-x_{k}\right). \end{array}$$

The Runge–Kutta method is used to generate prediction ${\tilde{x}}_{i,k}$ in DCKF. Namely, the solution of nonlinear propagation $X_{i,t,k}^{*}=f\left(X_{i,t,k-1}\right)$ is computed by the Runge–Kutta method.

**Figure 4:**
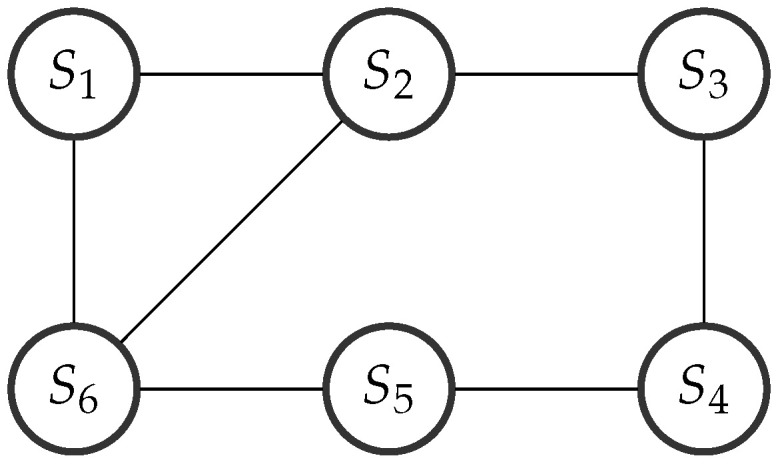
Topology of the network.

Simulation Case 1: We test the performance of the proposed DCKF, where the communication topology is fixed as shown in Figure 4. We assume each satellite can obtain both azimuth α and elevation β, where the measurement mapping is defined in (53) and (54). The measurement noise variances are generated by $R_{i}=idiag\left\{0.001^{2}\;\;0.001^{2}\right\},i=1,\ldots ,N$. The number of consensus is S=1.

**Figure 5:**
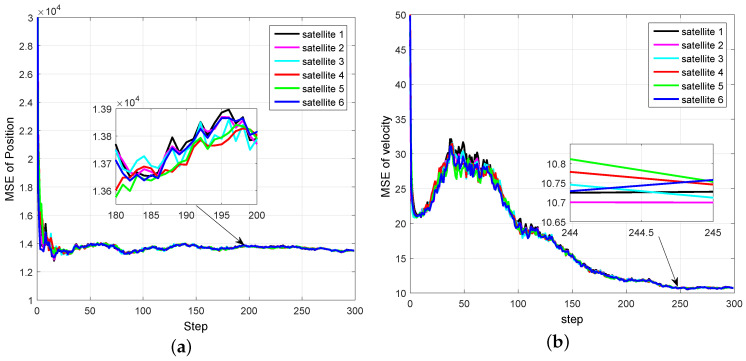
MSE curves of distributed cubature Kalman filter (DCKF) by different satellites under a fixed topology. (**a**) MSE of position; (**b**) MSE of velocity.

For Simulation Case 1, the comparison of MSE curves of different satellites is shown in Figure 5. The filtering precision and stability of the proposed DCKF for different satellites can be seen. It also illustrates that the estimations of different satellite almost reach a consensus, which can increase the robustness of tracking. More importantly, the estimate of each sensor is stable and converges even if the number of consensus is S=1, which can reduce the communication rate compared with DCIF in [26].

**Figure 6:**
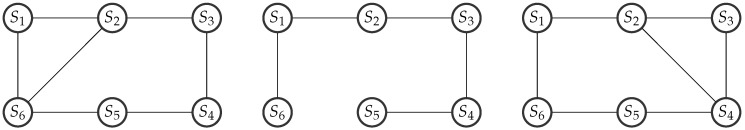
Switching topology of the network.

Simulation Case 2: In this case, we test the performance of the proposed DCKF for the switching topology, i.e., the communication is time-varying. To be specific, the communication topology is switching among the given topologies as shown in Figure 6. The setting of initialization and noise variances is the same as Simulation Case 1.

**Figure 7:**
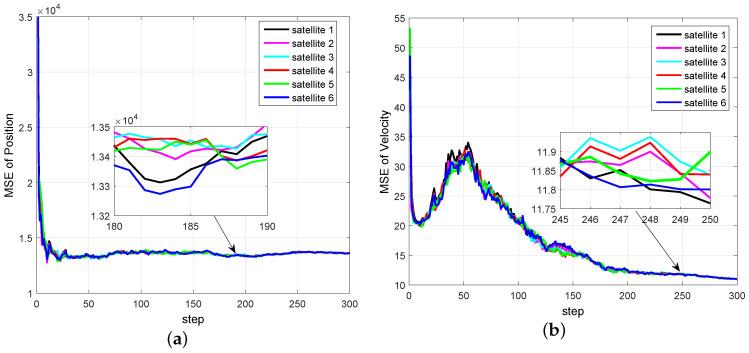
Distributed cubature information filter (DCIF) for the switching topology. (**a**) MSE of position; (**b**) MSE of velocity.

As shown in Figure 7, the filtering precision and stability of the proposed DCKF for different satellites are also demonstrated. It should be noted that, for the switching case, the estimate of each node only needs the information of its neighbors. However, the algorithm in [16] needs global information $\Delta_{\max}$ ($\Delta_{\max}=3,2,3$ in Figure 6), where $\Delta_{\max}=\max_{i}d_{i}$, $d_{i}$ is the degree of node *i*, which is not easy to obtain in time.

Simulation Case 3: In this case, we compare the performance of DCKF with the distributed extend Kalman filter (DEKF) in [40] and the distributed cubature information filter (DCIF) in [26]. The discrete model (52) is adopted for the time update in the DEKF algorithm.

We assume that each satellite can only obtain either azimuth α or elevation β for the tested filters. To be specific, Satellites 1, 3 and 5 can only obtain the azimuth α, and Satellites 2, 4 and 6 can only obtain the elevation β of the object. In this case, we assume the measurement noises of satellite *i* to be $v_{i,k}$ ~$N(0,10^{-4})$.

**Figure 8:**
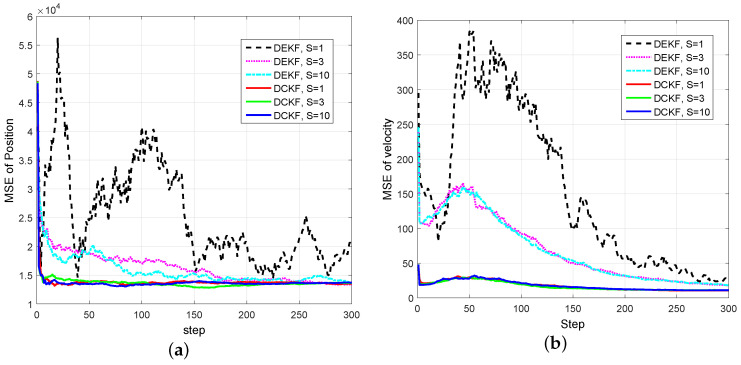
Comparison between DEKF and DCKF under weak observability for some sensors. (**a**) MSE of position; (**b**) MSE of velocity.

Figure 8 shows the comparison between DEKF and DCKF for different numbers of consensus. It can be seen that, for the cases S=1, S=3 and S=10, the DCKF performs more accurately than the DEKF, since the DEKF suffers form linearization errors due to numerically-linearizing the nonlinear dynamics and measurement map, which will enlarge the estimate errors. As *S* increases, the DEKF is more accurate. This is due to the fact that the local information will spread to the whole network as S→∞. Despite the weak observability, the proposed DCKF algorithm provides reasonable performance even for S=1, which indicates that the proposed DCKF is more robust and suitable for real-time applications with weak observability of some sensors than the DEKF.

**Figure 9:**
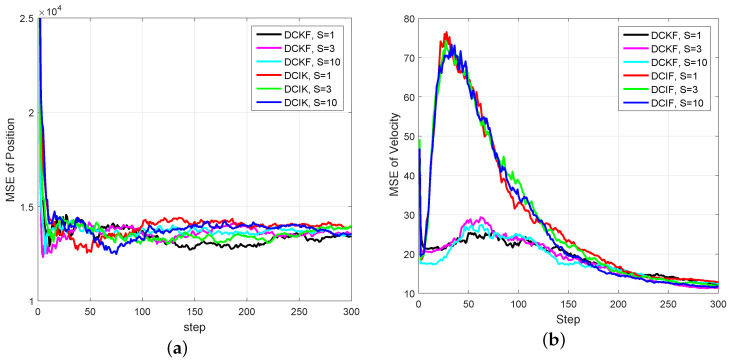
Comparison between DCIF and DCKF under weak observability for some sensors. (**a**) MSE of position; (**b**) MSE of velocity.

Figure 9 gives the comparison between DCIF and DCKF with different consensus numbers *S* under the weak observability condition. We also assume that Satellites 1, 3 and 5 can only obtain the azimuth α and Satellites 2, 4 and 6 can only obtain the elevation β of the object. It can be seen that, under the weak observability condition, all test filters can successfully track, and the estimation errors of the position of DCKF and DCIF are almost the same. However, there is a large velocity overshoot in DCIF, which indicates that the DCKF enjoys a stronger stability property than the DCIF.

The computational time of a single satellite of different filters is given in Table 3. All tests are operated on a notebook with an Intel central processing unit (i7 4510U) and MATLAB. Note that the computational time of the DCKF is longer than that of the DEKF, and the computational time of the DCIF is twice as long as the DCKF.

## 5. Discussion

The distributed Bayesian filter design has been researched, and a distributed cubature Kalman filter was proposed to deal with the time-varying topology and weak observability of sensors. It can be seen from Figure 2 and Figure 3 that the standard EKF cannot provide good results for weak observability of a single sensor. When it comes to the distributed setting, for the nodes with weak observability, both DCKF and DEKF can obtain stable estimation, and DCKF performs better than DEKF. From Figure 7, it also can be seen that DCKF is suitable for the case of switching topology. Namely, the proposed DCKF can handle the problem of blockage of the communication channel in time. Figure 9 illustrates that the proposed DCKF enjoys stronger stability properties than DCIF for the case of weak observability of some sensors, by noting that large velocity overshoot in the DCIF. A possible reason for such satisfactory performance of DCKF is that the global posterior PDF is considered for distributed estimation rather than just the innovations, as in DCIF. Moreover, the number of consensus in the DCKF could be one, which will conserve communication resources. However, we should highlight that the DCIF in [26] can approach the centralized solution as the number of consensus tends to infinity, and the DCKF in our paper cannot.

The K–L divergence for average consensus can be treated as a convex combination of the information matrices and vectors. This convex combination is well known as covariance intersection (CI) in the literature [41,42]. It is well known that the CI scheme provides an information fusion that is robust with respect to the unknown correlations among the information sources. The stability of such a fusion strategy for distributed estimation has been proven in [19] for linear time-invariant dynamics, and the results were extended to distributed EKF in [40].

A key point in our paper is that the global cost function (19) has a form of “sum-of-cost”, which is amenable to distributed implementations [32,43,44]. Information geometric optimization approaches can be used to construct the formulation, in which the natural gradient descent method is used to seek the optimal estimation. For example, in [29,30], the natural gradient descent method was used to construct the Bayesian nonlinear filter. In the distributed setting, a global optimal estimate can be obtained under the structure of the distributed convex optimization ([43]) by natural gradient descent.

In summary, the proposed DCKF has the advantages of strong stability and being more suitable for the cooperative object tracking problem compared to DEKF and DCIF. Future research issues mainly include the problems of measurement and communication delay, which will broaden the application of DCKF.

## 6. Conclusions

In this paper, we investigated the distributed Bayesian filter and proposed a distributed cubature Kalman filter. In order to solve the problems of weak observability and time-varying communication topology, we introduced Kullback–Leibler (K–L) divergence to measure the difference of local estimates, and the consensus estimate is achieved under the K–L average of local estimates. The simulation results indicate that, for the distributed space object tracking problem, the proposed DCKF has better results than DEKF and DCIF. Moreover, the proposed algorithm does not rely on the same measurement mapping of each sensor and can successfully track a space object for the time-varying communication topology and weak observability of some sensors.

## Figures and Tables

**Table 1:** Local estimation and consensus steps of DCIF.

Local estimation:
${\tilde{y}}_{i,k}={\tilde{P}}_{i,k}^{-1}{\tilde{x}}_{i,k}$, ${\tilde{Y}}_{i,k}={\tilde{P}}_{i,k}^{-1}$
$i_{i,k}\approx {\tilde{P}}_{i,k}^{-1}P_{i,xz,k}R_{i,k}^{-1}\left[\left(z_{i,k}-{\tilde{z}}_{i,k}\right)+P_{i,xz,k}^{⊤}{\left({\tilde{P}}_{i,k}^{-1}\right)}^{⊤}{\tilde{x}}_{i,k}\right]$,
$I_{i,k}\approx {\tilde{P}}_{i,k}^{-1}P_{i,xz,k}R_{i,k}^{-1}P_{i,xz,k}^{⊤}{\left({\tilde{P}}_{i,k}^{-1}\right)}^{⊤}$,
$y_{i,k}=\frac{{\tilde{y}}_{i,k}}{N}+i_{j,k}$, $Y_{i,k}=\frac{{\tilde{Y}}_{i,k}}{N}+I_{j,k}$;
**Consensus:**
**for s=1,…,S do**
$y_{i,k}^{s+1}=y_{i,k}^{s}-\epsilon \sum_{j\in N_{i}}\left(y_{i,k}^{s}-y_{j,k}^{s}\right)$,
$Y_{i,k}^{s+1}=Y_{i,k}^{s}-\epsilon \sum_{j\in N_{i}}\left(Y_{i,k}^{s}-Y_{j,k}^{s}\right)$,
**end for**
**Estimate of sensor *i*:**
$P_{i,k}={\left(NY_{i,k}^{S}\right)}^{-1},\;\;{\hat{x}}_{i,k}={\left(Y_{i,k}\right)}^{-1}{\left(y_{i,k}^{S}\right)}^{-1}$.

**Table 2:** Six orbital parameters of the observation satellites and object.

Six Orbital Parameter	$a_{h}$ (km)	*e*	*u* (Deg)	γ (Deg)	Γ (Deg)	$m_{h}$ (Deg)
Object	8667.13	0	73.9116	14.108	0	52.632
Satellite 1	9067.13	0	73.9116	128.495	0	52.942
Satellite 2	8067.1	0	73.9116	91.0768	0	18.88
Satellite 3	8667.13	0	73.9116	103.658	0	44.818
Satellite 4	8467.13	0	73.9116	116.24	0	70.756
Satellite 5	8267.13	0	73.9116	88.8216	0	96.694
Satellite 6	9067.13	0	73.9116	88.495	0	112.942

**Table 3:** Average computational time of the filters.

Filters	DCKF, S=1	DCKF, S=10	DEKF, S=1	DEKF, S=10	DCIF, S=1	DCIF, S=10
Time (s)	0.2002	0.2421	0.0428	0.0916	0.5087	0.5827
